# A comparison of tracheal intubation using intubrite laryngoscope and conventional MAC laryngoscope: An open, prospective, crossover manikin study

**DOI:** 10.1097/MD.0000000000035846

**Published:** 2023-11-10

**Authors:** Pawel Ratajczyk, Michal Fedorczak, Przemyslaw Kluj, Tomasz Gaszynski

**Affiliations:** a Department of Anesthesiology and Intensive Therapy, Medical University of Lodz, Poland.

**Keywords:** endotracheal intubation, intubrite laryngoscope, laryngoscopy, novice intubators

## Abstract

**Background:**

New devices are more available in the pre-hospital environment operational theaters and emergency departments. One is an intubrite laryngoscope (INT) with Dual LED lighting that combines ultraviolet and white LED. The study aimed to compare the efficacy of endotracheal intubation using INT and conventional laryngoscope performed by inexperienced paramedics (paramedics students) and paramedics with experience in advanced airways management in full and limited accessibility settings.

**Methods:**

It was an open, prospective, crossover manikin study. Sixty paramedics and paramedic students were recruited. Participants were divided into 2 equal groups depending on their experience (n = 30). Experienced participants were further randomly divided into 2 groups (n = 15). Inexperienced participants were also randomly divided into 2 groups (n = 15). The criterion of inexperience was 5 or fewer intubation by any laryngoscope. Inexperience participants were asked to perform tracheal intubation in standard pre-hospital settings (without limited access to manikin) (scenario A) and difficult pre-hospital settings (limited access to manikin - narrow space between benches) (scenario B). Experience participants were asked to intubate manikin in difficult pre-hospital settings.

**Results:**

In the normal pre-hospital environment, the success rate after the first attempt was 56,7% for conventional laryngoscope and 66,7% for intubrite. However, the overall effectiveness of tracheal intubation using both laryngoscopes in 3 attempts was 90% for both devices. The successful rate of first attempt intubation in a difficult environment by inexperienced was 73,3% for INT and 50% for conventional laryngoscope. Overall effectiveness was 83,3% and 86,7% respectively. The successful rate of first attempt intubation in the experienced group was 86,7% with INT compared to 60% with a conventional laryngoscope in difficult settings. Overall effectiveness was 96,7% for both devices.

**Conclusion:**

Intubrite provided better working conditions and make up for deficiencies in successful tracheal intubation by inexperienced participants in a normal and difficult environment. Tracheal intubation with intubrite was more effective in the experienced group. Tracheal intubation effectiveness with intubrite was also higher in the experienced group.

## 1. Introduction

Tracheal intubation performed in emergencies is more challenging than operating room intubation due to patient, operator, and environment-associated factors. The success rate is lower, the time needed to undertake the tracheal intubation is longer, and the complication rate is higher.^[1]^ Moreover, outside the operating theater, it leads to significant morbidity and mortality. Considerable experience is also required before one becomes proficient in direct laryngoscopy (DL) and tracheal intubation.^[2]^ Dynamically developing medical market propose new or improved equipment annually. New devices are more available in the pre-hospital environment operational theaters and emergency departments. One is an intubrite laryngoscope (INT) with Dual LED lighting that combines ultraviolet and white LED.

The study aimed to compare the efficacy of endotracheal intubation using INT and conventional laryngoscope performed by inexperienced paramedics (paramedics students) and paramedics with experience in advanced airways management in full and limited accessibility settings and to compare effectiveness of tracheal intubation in experienced and non-experienced group.

## 2. Methods

**Figure 1.**
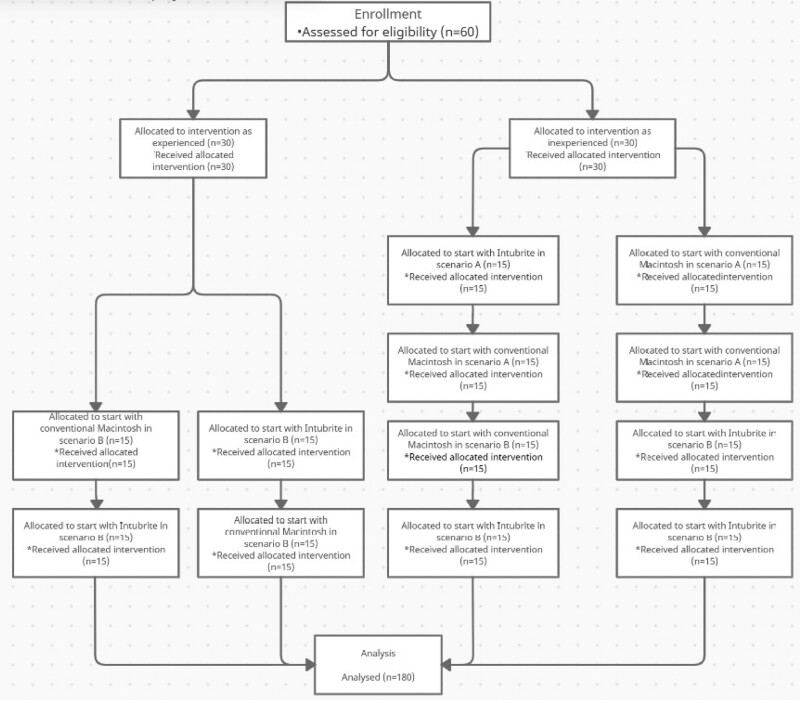
Flow diagram.

It was an open, prospective, crossover manikin study approved by the Local Ethics Committee of Medical University of Lodz, Poland (protocol number RNN/163/13/KB). Sixty paramedics and paramedic students were recruited. Participants were divided into 2 equal groups depending on their experience (n = 30). Experienced participants were further randomly divided into 2 groups (n = 15). Inexperienced participants were also randomly divided into 2 groups (n = 15). (Fig. 1) The criterion of inexperience was 5 or fewer intubation by any laryngoscope.

### 2.1. Simulation of the scenario

**Figure 2.**
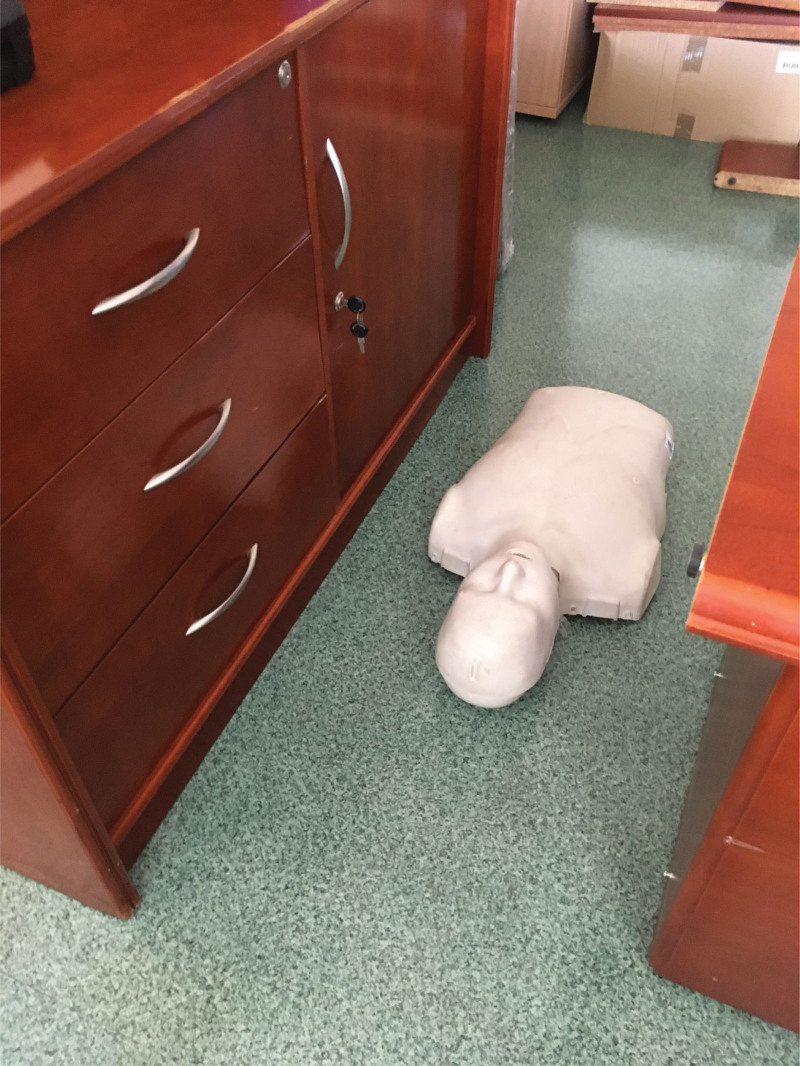
Difficult pre-hospital settings (limited access to manikin - narrow space between benches) (scenario B).

**Figure 3.**
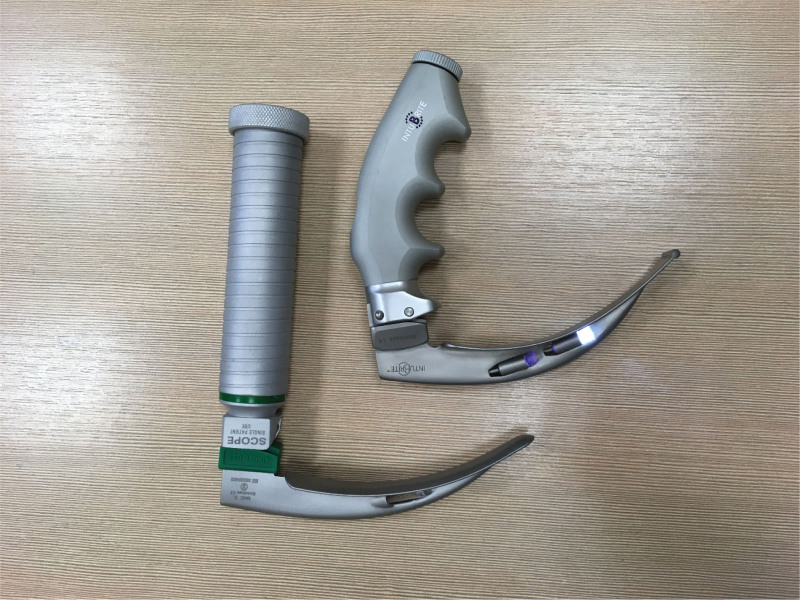
The laryngoscopes used for study: with Macintosh blade No. 3 (MAC; Mercury Medical, Clearwater, FL) and intubrite laryngoscope (INT; intubrite Llc, Vista, CA).

Inexperience participants were asked to perform tracheal intubation in standard pre-hospital settings (without limited access to manikin) (scenario A) and difficult pre-hospital settings (limited access to manikin - narrow space between benches) (scenario B) (Fig. 2). In standard prehospital settings, the manikin was placed on the ground in the middle of the simulation room without any access limitations. In difficult prehospital settings, the manikin was placed on the ground, between 2 benches (1 meter high), limiting access from both left and right sides (distanced 90 cm from the edges), making intubation more challenging. Experienced participants were asked to intubate manikin in difficult pre-hospital settings. Intubation attempts were limited to 30 seconds; up to 3 attempts were allowed. Successful intubation later than 30 seconds was recognized as an unsuccessful attempt. The participants were asked to perform tracheal intubation using an endotracheal tube 7.0 (Portex; Smiths Medical, Hythe, UK) with a conventional Macintosh laryngoscope with a size 3 blade (MAC; Mercury Medical, Clearwater, FL) or the INT, also with a Macintosh No. 3 blade (INT; intubrite Llc, Vista, CA) (Fig. 3). Each participant was randomly assigned to 3 tracheal intubation attempts with both devices in a different environment: The high-fidelity manikin was used for this study. Manikin (MegaCode Kelly Advanced, Laerdal, Norway).

### 2.2. Devices

Instructions on the correct use of the laryngoscope with Macintosh blade No. 3 (MAC; Mercury Medical, Clearwater, FL) and (INT; intubrite Llc, Vista, CA) were given before the procedure.^[3]^ The INT has dual LED lighting that combines ultraviolet and white LED to visualize the airway and handle with ergonomic shape (Fig. 3). The ultraviolet light causes phosphorus in the vocal cords and airway cartilage to glow in the dark and combines with a cool white LED for less glare. Conventional ID 7.0-mm tracheal tubes were used. The lubricant was applied on the tracheal tube. Resuscitator bag and 20 mL syringe were readily available and within range of the participants.^[4]^

### 2.3. Measurements and outcomes

The primary outcome was the success of the intubation attempt (i.e., tracheal or esophageal placement of the tube), which was recorded when the success of the ventilation attempt was confirmed by the manikin ventilation indicators.^[4]^ If intubation was unsuccessful, the participant has 2 more attempts. *Time to intubation was measured* from when the participant picked up the instrument until the tip had passed beyond the vocal cords.

## 3. Results

### 3.1. Scenario A

**Figure 4.**
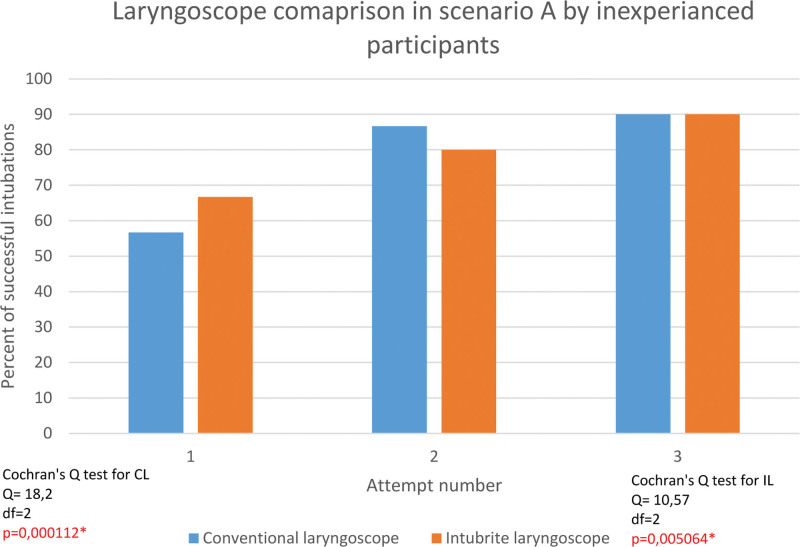
Intubation with conventional laryngoscope versus intubrite laryngoscope by inexperienced participants in normal pre-hospital.

In the normal pre-hospital environment, the success rate after the first attempt was 56,7% for conventional laryngoscope and 66,7% for intubrite. However, the overall effectiveness of tracheal intubation using both laryngoscopes in 3 attempts was 90% for both devices. (Fig. 4)

### 3.2. Scenario B

**Figure 5.**
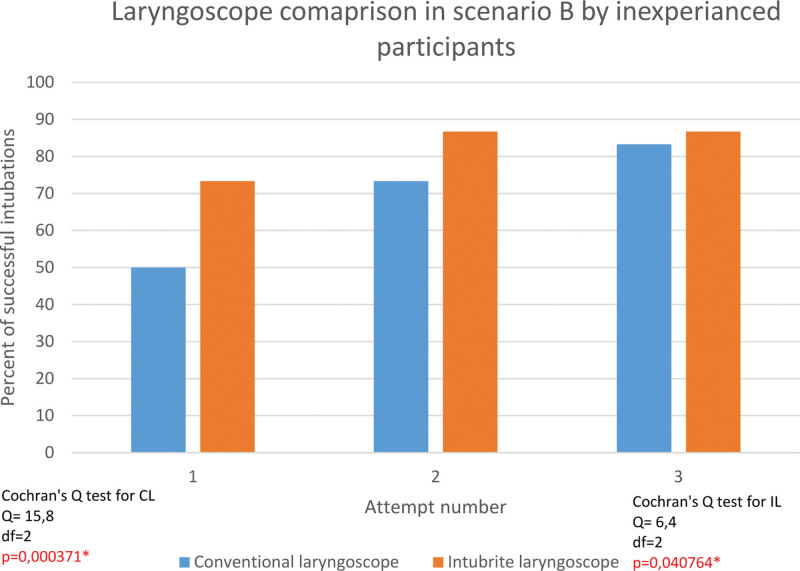
Intubation with conventional laryngoscope versus intubrite laryngoscope by inexperienced participants in difficult pre-hospital.

The successful rate of first attempt intubation in a difficult environment by inexperienced was 73,3% for INT and 50% for conventional laryngoscope. Overall effectiveness was 83,3% and 86,7%, respectively (Fig. 5).

**Figure 6.**
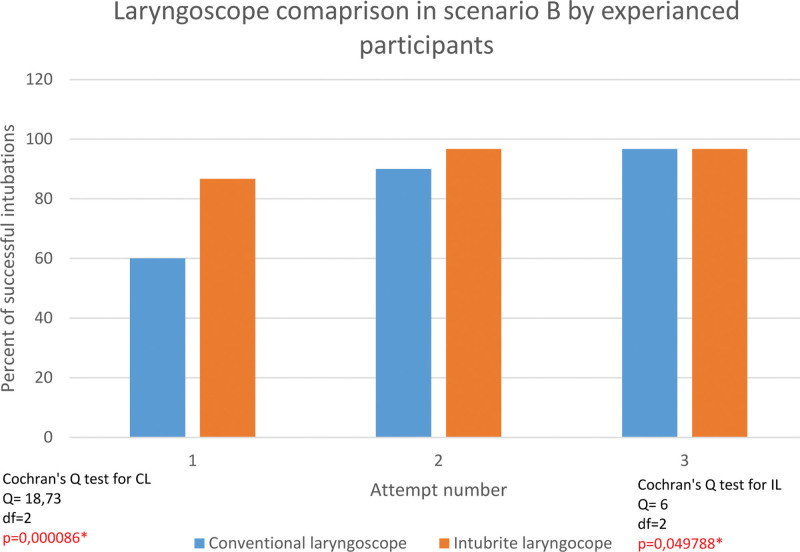
Intubation with conventional laryngoscope versus intubrite larngoscope by experienced participants in difficult pre-hospital settings.

The successful rate of first attempt intubation in the experienced group was 86,7% with INT compared to 60% with a conventional laryngoscope in difficult settings. Overall effectiveness was 96,7% for both devices (Fig. 6).

Such devices as intubrite provide better working conditions and make up for deficiencies in successful tracheal intubation by inexperienced participants in a normal and difficult environment where successful tracheal intubation is highly correlated with the number of attempts. Participants were more effective in the second and third attempts. The correlation was less significant in the intubrite group.

Tracheal intubation with intubrite was more effective in the experienced group. The first-attempt intubation effectiveness performed by skilled participants was higher with both devices. Like in the inexperienced group, successful intubation correlated with a number of attempts. The efficacy of tracheal intubation with intubrite was also higher in the experienced group.

## 4. Discussion

Since introduction of Macintosh and Miller laryngoscopes in 40s of 20th century the direct laryngoscope is the mostly used laryngoscope type enabling intubation of trachea.^[5]^ Skillful usage of it is a valuable skill of all providers working in both hospital and out-of-hospital care. However due to the necessity of obtaining straight line linking operator eyes with patient oral cavity and true glottis, intubation by means of direct laryngoscopes is a complex and technically fairly difficult procedure.^[6]^ According to literature, Macintosh laryngoscope intubation success rate is only 50% in case of use by an operator without clinical experience. It increases to the level of about 90% after execution of at least 50 intubations.^[7]^ Similar data is not available for Miller laryngoscope. Also teaching trachea intubation with those laryngoscopes with the vision line techniques is hard, because a teacher cannot share airways’ images with a student.^[5]^ Over the years many variants of direct laryngoscopes with following blades were developed: Miller (1941), Macintosh (1943), Soper (1947), Jackson-Wisconsin (1952), Bizzari-Guffrid (1958), Bainton (1987), and recently VieScope laryngoscope, but until now primarily used remained the oldest 2: Macintosh and Miller, with vast majority of Macintosh blade.^[8,9]^ Nowadays its role in difficult airways visualization in adult patients is largely limited to Macintosh and MacCoy laryngoscopes.^[5]^ McCoy laryngoscope which was introduced into clinical practice in 1993 is the laryngoscope with Macintosh like blade and the tip which bends upon pressing the lever to further lift the epiglottis. The mechanism of levering the tip is crucial for the defined and consistent performance of the blade.

The INT introduced in 2008, which has Macintosh like blade, has changed the lighting method used—the commonly used white light is replaced by a system of 2 different light sources: ultraviolet light and light emitting diode type light. This type of lighting increases the image contrast and reduces glare on wet mucous membranes during DL. The laryngoscope handle is made from aluminum, thereby making it lightweight, and the ergonomic shape with finger embossing makes the laryngoscope comfortable and intuitive to use. The arched profile of the handle allows for improved maneuvering during the intubation procedure which is important for less experienced operators.

Airway management in the limited access situation creates a unique set of problems for intubation providers. Performance of basic and advanced airway maneuvers may become burdensome or near impossible using conventional approaches. The example of limited access to patient when performing airway management is intubation of patient entrapped in the vehicle. An intubation of a patient confined to a car driver seat ought to be immediately performed as long as evacuation is impossible and protection of the airways is a must.^[9]^ The airway management in entrapment-trauma is challenging due to restricted access, the seated position of the patient and the need for cervical spinal immobilization.^[10]^

There is no many papers on advanced airways in entrapped patients. Those available are performed on group of experienced doctors: anesthesiologists^[10–12]^ or emergency physicians.^[9,13]^ The results of these studies show, that devices like videolaryngoscopes,^[10]^ optical laryngoscopes^[12]^ or supraglottic devices^[11]^ are superior to standard MAC in this conditions with the exception of study of Wetsh et al who proved that experienced anesthetists had more success in entrapped patients with MCL.^[12]^ There are some papers evaluating intubation performed by paramedics in simuated conditions of patient entrapped in the vehicle.^[9,14]^ In a paper of Domian et al a 34 participants (24 paramedics and 8 physicians) with diverse work experience were asked to intubate a manikin placed on a driver seat in rigid collar immobilization. Participants were located either in front of the manikin, at the back of the manikin—behind the manikin head or from the manikin side. Intubation attempts carried out on a manikin by participants were performed with the use of 4 devices: laryngoscope with Macintosh blade, laryngoscope with TruView Evo1 blade, optical laryngoscope Airtraq and intubating larynegal mask airway–fastrach (ILMA-FastTrach).^[9]^ In this paper the highest efficiency during intubation was achieved when ILMA -Fastrach system. The physicians were more effective than paramedics in utilization of Macintosh and TruView systems. All participants in this study had clinical experience with intubation. Another paper evaluating paramedics intubation success rate in entrapped patients revealed that ILMA is superior to laryngoscopes including videolaryngoscopes (AirTraq and KingVision).^[14]^ Asai performed a similar to our study of intubation using MCL and Pentax AWS in manikin simulating patient entrapped in car.^[15]^ He found out that both visualization of the entrance to the larynx and intubation using Pentax AWS was significantly faster and more effective than MCL. Other studies were performed in manikins with only limited head access: manikin was placed near the wall^[10,11]^ except for Wetch, who placed the manikin in the real car.^[12]^

In the inexperienced group, the INT resulted in a lower correlation of intubation success with the number of attempts. Although tracheal intubation with the classic and INTs, the number of attempts correlated significantly with efficacy, this correlation level was much lower when inexperienced individuals used the INT.

This means that intubrite allowed a reduction in the number of attempts to achieve successful intubation. An even stronger tendency in this aspect was noted for difficult conditions. The significance level (assessing the correlation between the number of attempts made and successful intubations) decreased dramatically with intubrite intubation - compared to the classic laryngoscope. This shows that in the inexperienced group in difficult conditions, the validity of using the intubrite is even higher than during normal conditions.

An analogous relationship was also demonstrated concerning experienced participants. Also, in their case, the level of effectiveness became significantly less correlated with the number of attempts performed when they worked with the INT under difficult conditions.

Although emergency intubation remains the gold standard for airways management, it is also associated with the risk of complications.

The above circumstances increase the risk of complications if the intubating personnel is not experienced.^[16]^ Our study demonstrated that the INT significantly reduces the correlation between the number of attempts and final successful intubation. This is important as each intubation attempt increases the risk of complications.

Similar conclusions are presented by Szirer, who emphasizes that the risk of complications increases, particularly in difficult intubation performed in the out-hospital setting.^[17]^ Therefore, it seems particularly important that - as it was demonstrated - the INT is significantly more effective under difficult conditions, mainly when used by experienced personnel. This group achieved significantly higher intubation success on the first attempt.

Our study shows that less experienced rescuers achieve the same efficiency when working with intubrite as professional rescuers with conventional laryngoscopes. Moreover, it has been proven that under difficult conditions, intubrite is as effective as a conventional laryngoscope under normal conditions.

There are no other studies evaluating the use of intubrite videolaryngoscope by inexperienced intubators. Therefore we can compare results to those of other researchers performing studies on other videolaryngoscopes.

Introducing new devices for clinical practice allows for better intubation performance by novice medical personnel. In the simulation study of O`Carroll et al novice intubators performed intubation with 4 different devices: standard laryngoscope, videolaryngoscope, AirTraq optical laryngoscope, and fiberoptic laryngoscope.^[18]^ The use of a videolaryngoscope was the most efficient and effective. Furthermore, the authors observed a correlation between playing with videogames of intubators and success ratio and self-confidence when using a videolaryngoscope.

Rya et al compared McGrath videolaryngoscope and standard laryngoscope in the hands of inexperienced intubators.^[19]^ They evaluated the performance of 25 medical students with no previous experience performing tracheal intubation using an easy intubation scenario in a manikin. The order of device use was randomized for each student. They found out that in the case of the use of videolaryngosope first pass success ratio is significantly higher compared to standard laryngoscope. Novices achieved a higher overall rate of successful tracheal intubation, avoided esophageal intubation, and produced less dental trauma when using the McGrath. The view at laryngoscopy was significantly better with the McGrath. Intubation times were similar for both laryngoscopes and became shorter with practice.

The question is if manikin studies reveal a tendency that could be seen in actual patients. In the study of Shih et al, researchers evaluated intubation performed by novice intubators in patients using videolaryngoscope.^[20]^ They observed that the overall first-shot success rate was 81.3%. For inexperienced doctors, videolaryngoscopy (VL) produces high first-shot success rates for tracheal intubation.

In the study by twenty volunteers, who had had only manikin training for tracheal intubation and attempted 5 intubations with either technique in patients scheduled for general anesthesia, Nouruzi-Sedeh et al evaluated the use of videolaryngoscope in 200 patients.^[21]^ The overall success rate was 93% for the GlideScope technique versus 51% for DL. This study is proof that videolaryngoscope in the hands of inexperienced intubators can be an excellent alternative to standard laryngoscope.

Another question is skill retention. In the study of Ghotbaldinian et al researchers evaluated to what extent recently acquired basic skills of endotracheal intubation, based on DL or VL, are being maintained over time by inexperienced operators after 3 months of intubation training.^[22]^ No significant differences in skills retention were found between DL and VL regarding the time for successful intubation or the number of adverse events. However, the first intubation was significantly slower regardless of the technique, but, the use of a standard laryngoscope was slower and associated with more incidents of esophageal intubation and dental manipulation than was with a videolaryngoscope. In the study of Ozge et al in cross-sectional study, the manikin neck collar and spine board created a complicated airway model with cervical immobilization inexperienced paramedic students intubated manikin with either videolaryngoscope or standard MAC.^[23]^ All volunteers had previous intubation experience with DL but not with video laryngoscopy. There was a statistically significant difference in the first-attempt success rates of the procedure between the groups in favor of video laryngoscope. This study confirms that non-experienced intubators can quickly learn new devices. The learning curve of intubation using videolaryngoscopes was described by Declerqc et al^[24]^ This study demonstrates that DL needs prolonged learning, whereas videolaryngoscopes require less operator skill than DL to effectively and rapidly secure the airway.

There is one study comparing INT with MAC in manikin settings, but the intubation was performed under conditions simulating those in non-hospital conditions, - the manikin was placed at the floor level without limited access to patient.^[25]^ The use of the INT by people with no clinical experience shortens the time of intubation and improves the laryngeal view compared to the standard Macintosh laryngoscope. Moreover, in cases when the INT is used in simulated out-of-hospital conditions, the percentage of repeated intubation attempts and tooth damage is lower compared to the Macintosh laryngoscope; the results are similar to those of J. Tesler and J. Rucker.^[26]^ Similar results were obtained by Tomasz M. Gaszyński, who stated that the INT is less traumatic to patients than the Macintosh laryngoscope.^[27]^ On the other hand, Szarpak and coworkers found that in difficult airways (tongue edema), the time needed for intubation by novice physicians in manikin tests was similar for both laryngoscopes, although the effectiveness of the first intubation was higher for Macintosh laryngoscopes.^[3]^

### 4.1. Study limitations

The main limitation of the study is that it was a manikin study. The evaluation of the performance of intubation with IntubBrite laryngoscope by novice intubators would be more adequate in patients. However, the manikin studies can be the introduction to more advanced studies performed on patients.

## 5. Conclusion

IntuBrite provided better working conditions and make up for deficiencies in successful tracheal intubation by inexperienced participants in a normal and difficult environment. Tracheal intubation with intubrite was more effective in the experienced group. Tracheal intubation effectiveness with intubrite was also higher in the experienced group.

## Author contributions

**Conceptualization:** Tomasz Gaszynski.

**Data curation:** Michal Fedorczak, Przemyslaw Kluj.

**Formal analysis:** Michal Fedorczak, Przemyslaw Kluj.

**Investigation:** Michal Fedorczak, Przemyslaw Kluj.

**Methodology:** Tomasz Gaszynski.

**Project administration:** Tomasz Gaszynski.

**Supervision:** Tomasz Gaszynski.

**Writing – original draft:** Michal Fedorczak.

**Writing – review & editing:** Pawel Ratajczyk, Tomasz Gaszynski.
